# Prevalence of Dentinal Hypersensitivity among Dental Patients Visiting Tertiary Care Center: An Observational Study

**DOI:** 10.31729/jnma.8881

**Published:** 2025-02-28

**Authors:** Saru Ghimire, Sagar Ghimire, Samarika Dahal, Sirjana Dahal, Neetika Paudel, Prayash Paudel

**Affiliations:** 1 Maharajgunj Medical Campus, Maharajgunj, Kathmandu, Nepal; 2 Department of Radio Diagnosis and Imaging, Post Graduate Institute of Medical Research, Sector 43, Chandigarh, India; 3 Department of Oral Pathology and Forensic Dentistry, Maharajgunj Medical Campus, Maharajgunj, Kathmandu, Nepal; 4 Department of Public Health Dentistry, Maharajgunj Medical Campus, Tribhuvan University, Maharajgunj, Kathmandu, Nepal

**Keywords:** *dentinal hypersensitivity*, *prevalence*, *tertiary care center*

## Abstract

**Introduction:**

Dentinal hypersensitivity is a widespread condition characterized by a brief, sharp pain arising from exposed dentin in response to external stimuli, which cannot be linked to any other dental conditions. This study aimed to estimate the prevalence of dentinal hypersensitivity among patients attending a tertiary health care center.

**Methods:**

A observational cross-section study was conducted, involving 376 patients aged 10-70 years who visited a tertiary care center. Convenience sampling was employed to select participants, and the prevalence of dentinal hypersensitivity was assessed through a questionnaire. Data were collected from the Department of Oral Medicine and Radiology after obtaining ethical approval Institutional Review Committee (Reference number: 385 (6-11) E2 079/80). The analysis was performed using Microsoft Excel, and descriptive statistics were reported .

**Results:**

There were 376 patients in the study and the prevlence of dentin hypersensitivity wasa 236 (62.77%; CI 95%: 57.66%-67.67%). Among those diagnosed, 138 (73.02%) were male, and 98 (52.41%) were female. The age-specific prevalence was found to be 30 (50.85%) in patients aged 10-19 years, 73 (63.48%) in those aged 20-29 years, 42 (53.16%) in patients aged 30-39 years, 23 (51.11%) in those aged 40-49 years.

**Conclusions:**

This study identified a high prevalence of dentin hypersensitivity among the patient population, with variations observed across different age and sex groups.

## INTRODUCTION

Dentinal hypersensitivity (DH) is a condition commonly encountered in dental practice, parTicularly in individuals who suffer from abrasion, recession of The gingiva, and Tooth erosion. DH causes patient discomfort and hampers everyday activities. It is distinguished by sharp, stabbing pain caused by exposed cervical dentin.^1^

Dentinal hypersensitivity pain intensifies due To patent dentinal Tubules responding To various stimuli like temperature changes, touch, osmotic, chemical, or dispersal, which cannot be attributed to other conditions.^2^ Dentinal hypersensitivity frequently impacts adults, particularly those in their 30s, though it can affect individuals aged 29 To 49 years. Prevalent in up To 47% of The general population,^3^ it is even more common among periodontal patients. Dental hypersensitivity often affects The mucogingival areas of The canine- and premolar-sextant anterior locations prone To gingival recession.^4,5^

This study aimed To find The prevalence of dentinal hypersensitivity among The patients visiting The Tertiary health care center.

## METHODS

This observational cross-section study was conducted at Tribhuvan University Dental Teaching Hospital, located in Maharajgunj, Kathmandu. The data collection period extended from February to May 2023. Ethical approval for this research was obtained from the Institutional Review Committee (IRC) of Tribhuvan University Dental Teaching Hospital [Reference number: 385 (6-11) E2 079/80]. Participants in the study were selected from patients visiting the department during the study period. Each participant provided written informed consent, ensuring their voluntary and informed participation. For minors involved in the study, assent was obtained along with informed consent from their legal guardians.

Patients with severe and complex dental conditions— such as extensive dental caries, advanced periodontal disease, or significant oral lesions were excluded from participation. Individuals who were taking medications known to affect dental sensitivity, such as antihypertensives, antidepressants, antihistamines, and anticonvulsants were also excluded from the study. Furthermore, pregnant women were excluded from the study due to the physiological changes they experience, which can temporarily influence dental sensitivity. Additionally, individuals who had recently undergone dental procedures such as tooth extractions, scaling, or root canal treatments were excluded.

Data collection was performed using a questionnaire. The questionnaire was based on a previously validated questionnaire used in the United Kingdom related to the understanding of DH.^6-8^ and has been recently updated and used in several studies namely in Brazil, India, and Kuwait.^9-11^

The sample size was calculated using following formula:


$$\begin{array}{l} \begin{array}{ll} \text{n} & ={\text{Z}}^{2}\times \frac{\text{p}\times \text{q}}{{\text{e}}^{\text{2}}}\\ & =1.96^{2}\times \frac{0.57\times (1-0.57)}{0.05^{2}} \end{array}\\ \text{n }=376 \end{array}$$

Where:

Z represents the z-value (1.96) at a 95% Confidence Interval (CI).
P is the estimated prevalence of DH in the general population (57%).^12^
q = 1-p
e is the margin of error (5%).

The calculated minimum sample size was 376 participants. The sample was selected using convenience sampling, where participants were chosen based on their availability and willingness to participate.

Once data were collected, they were systematically entered into Microsoft Excel 2019 for analysis.

## RESULTS

There were 376 patients included in the study, out of which 189 (50.27) were male and 115 (30.59%) were in the age group of 20-29 years. The DH was observed in 236 (62.76%) of the patients. The proportion of male and female having DH was 138 (73.02%) and 98(52.41%) respectively. All patient 24 (100%) in the age group 60-70 years had DH (Table 1).

**Table 1:** Age wise frequency distribution of total population and the DH rate in each age group (N=376, n = 236)

Age	Frequency in total population N(%)	Proportion in age group having DH, n(%)
10-19	59(15.69)	30 (50.85)
20-29	115(30.59)	73 (63.48)
30-39	79(21.01)	42 (53.16)
40-49	45(11.97)	23 (51.11)
50-59	54(14.36)	44 (81.48)
60-70	24 (6.48)	24 (100%)

DH = Dentinal Hypersensitivity; N=Total population; n = Population with Dentinal Hypersensitivity

Among those diagnosed with hypersensitivity, 223 (94.49%) reported sensitivity to sweet food consumption, and 219 (92.8%) experienced discomfort from hot or cold foods (Table 2).

**Table 2:** Frequency of Dental Hypersensitivity as per the etiological factors (n= 236).^*^

Etiology	n (%)
Hypersensitivity to sweet consumption	223 (94.49 )
Hypersensitivity to hot/cold food	219 (92.8 )
Hypersensitivity to breathing with gritted teeth	166 (70.34)
Hypersensitivity to vigorous tooth brushing	104 (44.07 )

* Multiple Response Question DH = Dentinal Hypersensitivity

## DISCUSSION

The prevalence of dentinal hypersensitivity was 62.76%, which is substantially higher than the data presented in a study conducted by Kanehira et. al, where a similar setting was used, and the prevalence was reported to be much lower.^13^ The disparity between the two studies could be attributed to several factors, such as differences in sample selection, methodologies, and diagnostic tools used. The value we observed is also significantly higher than the prevalence reported in a study conducted in the United Kingdom, where the prevalence was found to be only 4.1%. This stark difference can likely be explained by the methodologies employed in the UK study, which used a combination of questionnaire-based surveys followed by clinical examination. In contrast, our study setting did not incorporate clinical validation to the same extent, despite having a similar sample size.^14^ Such discrepancies in data highlight the importance of research methods in accurately gauging the prevalence of dentinal hypersensitivity.

Similarly, a study conducted in India reported a lower prevalence of 25%, which contrasts with our study's higher percentage.^15^ This variation in prevalence between studies can likely be attributed to differences in population demographics, environmental factors, and the types of research methods used. For instance, the Indian study may have employed more stringent criteria for diagnosing hypersensitivity or could have studied a population with different dietary and oral hygiene habits. In contrast, our study was conducted in a tertiary care center, where patients may already exhibit a higher degree of dental health issues, potentially leading to an inflated prevalence rate. Additionally, the use of clinical examinations versus self-reported data may have contributed to the variation.

In yet another study conducted in Turkey, the reported prevalence was 5.3%, which is also much lower than the result from our study.^16^ The varying prevalence rates across different geographical regions could be due to several factors such as differences in dietary habits, dental hygiene practices, social and economic factors, and perhaps even genetic predispositions. Diverse research has consistently shown that dentinal hypersensitivity appears in various forms and distributions depending on the population being studied.^17^ These variations highlight the need for standardized research methods across studies to enable more accurate comparisons. The differences in customs, diets, and techniques used to detect dentinal hypersensitivity are crucial contributing factors to the varied prevalence observed worldwide. Moreover, geographical variation and the difference in social classes of subjects involved in research can also lead to such disparities in results.^3^

Our study further examined the most common etiological factors contributing to dentinal hypersensitivity. We found that sweet food consumption was the most prevalent cause, reported by 94.7% of the sample population as the primary stimulus for their hypersensitivity. The link between sweet foods and hypersensitivity may be due to increased tooth decay, enamel erosion, and exposure of dentinal tubules. Cold and hot stimuli were also reported as a significant cause in 93% of the sample population. This result aligns with the widely accepted understanding that temperature changes often exacerbate hypersensitivity in exposed dentin areas.

Vigorous tooth brushing, another commonly cited cause, was found to be a contributing factor in 44% of the sample population. Excessive brushing can lead to enamel wear and gingival recession, both of which expose dentin and make teeth more susceptible to sensitivity. Comparatively, a study conducted in Nigeria found that cold or heat stimuli were the leading causes in 35% of their population, which is lower than our findings.^18^ Additionally, the Nigerian study also identified air stimulation by sour or sweet stimuli as a contributing factor, adding another layer of complexity to the different ways in which sensitivity can manifest across populations.

Interestingly, the result of our study was somewhat consistent with findings from a study conducted in the United Kingdom, where cold drinks were identified as the most common cause of hypersensitivity.^19,20^ Cold drinks were also the second most prevalent cause in our study, suggesting a possible cross-cultural similarity in how cold stimuli affect dental sensitivity. The prevalence of dentinal hypersensitivity due to vigorous tooth brushing in the UK study was 37%, which is slightly lower than our finding of 44%.^19^ The consistent identification of vigorous brushing across studies highlights the importance of educating patients on proper brushing techniques to prevent unnecessary enamel wear.

Moreover, the UK study also noted smoking as another common cause of hypersensitivity.^20^ Smoking is known to contribute to periodontal disease and gingival recession, both of which can expose dentin and lead to hypersensitivity. However, our study did not find any significant correlation between smoking and dentinal hypersensitivity. This may be due to differences in the population demographics or the fact that smoking rates in our sample were relatively low, potentially skewing the results.

Another study conducted in Turkey identified gingival recession as the most common cause of dentinal hypersensitivity.^16^ This finding is particularly relevant, as gingival recession can expose the tooth root, making it more susceptible to sensitivity. However, gingival recession was not recorded as a major factor in our study, which could be due to differences in the age distribution of the populations studied or the fact that we did not specifically focus on periodontal health as a contributing factor.

Regarding the age distribution of dentinal hypersensitivity, our study found that the highest prevalence was among The 60-69-yea r-old age group, with a prevalence of 100%, followed by the 50-59-year-old group at 81.48%. This was followed by the 20-29-year-old group with a prevalence of 63.48%, the 30-39-year-old group at 53.16%, and finally, the 40-49-year-old group with a prevalence of 51.11%. Interestingly, the youngest age group, 10-19 years, also had a significant prevalence rate of 50.85%. These findings contrast with Those of Rees et al., who reported the greatest incidence of hypersensitivity in the 30-49-year-old age group, followed by the 40-49-year-old group.^3^ The age-related differences in hypersensitivity prevalence could be attributed to various factors, including the accumulation of dental wear and tear over time, dietary changes, and differences in oral hygiene habits across different age groups. Additionally, the sharp increase in prevalence in the older age groups (50-59 and 60-69 years) could reflect the impact of long-term dental health deterioration, possibly related to factors such as enamel erosion and gingival recession.

A similar study conducted by Dhaliwal et al. in India also found That The peak prevalence of hypersensitivity was in the 50-59-year-old age group.^15^ This consistency with our findings further supports the idea that older individuals are more likely to experience dentinal hypersensitivity due to the natural aging process and its impact on dental health. Furthermore, a study conducted by Liu et al. also identified the 50-59-year-old group as having the highest prevalence, which reinforces the notion that older age groups tend to experience higher rates of dentinal hypersensitivity. The Canadian Advisory Board on Dentine Hypersensitivity has also indicated that the prevalence of hypersensitivity among young individuals is often underreported. They recommend that future research should focus on identifying the associated risk factors and implementing appropriate care strategies for younger populations, who may not seek treatment as frequently as older individuals.^15^ The relatively high prevalence we observed in the younger age groups, particularly the 20-29-year-old group (63.48%), suggests that this demographic might not be as unaffected by hypersensitivity as previously Thought, emphasizing The need for Targeted awareness and intervention strategies for This age group.

Another interesting finding from our study was The gender distribution of dentinal hypersensitivity. We found that the prevalence was higher among males, at 73.02%, compared to females, who had a prevalence of 52.41%. This contrasts with several other studies that reported higher prevalence rates among females.^15^ For instance, one study observed that the prevalence of hypersensitivity among females was significantly higher, suggesting that women may be more likely to report symptoms of hypersensitivity or may have different oral health care practices compared to men.^21^

The discrepancy in our findings, where males exhibit a higher prevalence, could be due To a variety of factors, such as differences in dietary habits, oral hygiene practices, or levels of dental care awareness between men and women in The population studied. Cultural, social, and biological factors often influence The gender differences in The prevalence of dental conditions like hypersensitivity. Further research is needed To clarify These discrepancies, as well as To explore whether males in certain populations may be less likely to seek treatment until symptoms become more severe, thereby inflating the observed prevalence rates in males in our study.

It is important to note that our study was crosssectional in nature, which introduces certain limitations. Cross-sectional studies provide a snapshot of a particular population at one point in time, making it difficult To draw conclusions about changes or Trends over Time. Moreover, There is a possibility of selection bias and information bias in our study. Since we employed a convenience sampling method, the study was conducted in a single center with a relatively small sample size. As a result, the findings may not be generalizable to a larger population, and future studies should aim to include a more diverse and representative sample.

In addition to these limitations, our study did not take into account several socio-economic factors that could influence the prevalence of dentinal hypersensitivity. Factors such as income, education level, and access to dental care may all play a role in the development and reporting of hypersensitivity, and these variables should be considered in future research. Furthermore, we recommend that future studies adopt more rigorous methodologies, including The use of intraoral examinations in conjunction with questionnaires, to ensure that the results are not overstated and that a more accurate assessment of hypersensitivity prevalence is obtained.

## CONCLUSIONS

Our study highlights The high prevalence of dentinal hypersensitivity in the population studied, with sweet food consumption, temperature changes, and vigorous brushing being the most common contributing factors.
